# Mathematics and Relatedness: Predicting Optimal Social Engagement in Late Life

**DOI:** 10.1093/geroni/igaa057.1275

**Published:** 2020-12-16

**Authors:** Christine Williams, Emmanuelle Tognoli, Christopher Beetle

**Affiliations:** Florida Atlantic University, Boca Raton, Florida, United States

## Abstract

Multiple causes converge for older adults to shed social relationships. Lost opportunities for social engagement are tied to weakened cognitive reserve and under-optimal aging in health and disease. For example, a woman, 75, regularly strolls with younger friends. At 80, her reduced motor fitness makes it hard to keep pace and she withdraws her participation. With same-age peers, she might continue this healthy physical and social activity a few more years by unobtrusively shortening the outing or by slowing her pace. A man, 85, loves to debate politics with family, but his turn at talks diminish: his hearing loss (sensory) prevents quick grasp of the discussion; his slower verbal fluency (cognitive) hamper quick-witted replies. Both examples illustrate that social aging is not only a ¬¬¬property of the aging individual. Social context plays an important role. Our recently formed interdisciplinary group (geropsychiatric nurse, mathematical physicist and complexity scientist) is studying the systemic complexities of social aging with experiments and mathematical models. Our aim is to present our model and aging-focused hypotheses, as well as empirical validation in younger adults. Four key variables are group size and heterogeneity, and the strength and adaptability of social coordination. Our current results show that people coordinate better with others like them in pace, but they lose the ability to coordinate with people whose pace is different. We anticipate that our program of research will deliver evidence-based recommendations on social-engineering of activities that maximize opportunities for sustained interactions among older adults.

